# IVIG plus Glucocorticoids versus IVIG Alone in Multisystem Inflammatory Syndrome in Children (MIS-C) Associated with COVID-19: A Systematic Review and Meta-Analysis

**DOI:** 10.1155/2022/9458653

**Published:** 2022-03-29

**Authors:** Robin Rauniyar, Aman Mishra, Sanjeev Kharel, Subarna Giri, Rohit Rauniyar, Shikha Yadav, Gajendra Chaudhary

**Affiliations:** ^1^Maharajgunj Medical Campus, Tribhuvan University, Institute of Medicine, Kathmandu, Nepal; ^2^Internal Medicine, McLaren Flint/Michigan State University (MSU), Flint, MI, USA; ^3^Nepalgunj Medical College, Kathmandu University, Dhulikhel 45200, Nepal

## Abstract

**Background:**

There is limited information available regarding the management of multisystem inflammatory syndrome in children (MIS-C) associated with SARS-CoV-2. We performed a systematic review and meta-analysis to evaluate the optimal treatment using IVIG alone versus IVIG plus glucocorticoids.

**Methods:**

PubMed, Google Scholar, EMBASE, and Cochrane databases were searched along with other secondary searches. Studies published within the time frame of January 2020 to August 2021 were included. We screened records, extracted data, and assessed the quality of the studies using NOS. Studies that directly compare the two treatment groups were included. Analyses were conducted using the random-effects model (DerSimonian-Laird analysis) if *I*^2^ > 50% and fixed-effects model was used if *I*^2^ < 50%.

**Results:**

We included three studies in the final quantitative analysis. The initial therapy with the IVIG plus glucocorticoids group significantly lowered the risk of treatment failure (OR 0.57, 95% CI (0.42, 0.79), *I*^2^ 45.36%) and the need for adjunctive immunomodulatory therapy (OR 0.27, 95% CI (0.20, 0.37), *I*^2^ 0.0%). The combination therapy showed no significant reduction in occurrence of left ventricular dysfunction (OR 0.79, 95% CI (0.34, 1.87), *I*^2^ 58.44%) and the need for inotropic support (OR 0.83, 95% CI (0.35, 1.99), *I*^2^ 75.40%).

**Conclusion:**

This study supports the use of IVIG with glucocorticoids compared to IVIG alone, as the combination therapy significantly lowered the risk of treatment failure and the need for adjunctive immunomodulatory therapy.

## 1. Introduction

Severe acute respiratory syndrome coronavirus 2 (SARS-CoV-2) responsible for coronavirus disease 2019 (COVID-19) led to a pandemic affecting people of all ages. Children and adolescents were affected in much smaller numbers and experienced mild symptoms that do not require medical intervention. However, two to four weeks after being tested positive for COVID-19, a significant number of children developed a severe inflammatory condition referred to as multisystem inflammatory syndrome in children (MIS-C) associated with SARS-CoV-2 [1–3]. It was first reported in April 2020 by clinicians in the United Kingdom when eight previously healthy children presented with fever and cardiovascular shock, preceded a surge in the number of cases of children with similar presentations worldwide [1, 2].

Although MIS-C is uncommon, it has led to serious and potentially life-threatening illnesses in previously healthy children and adolescents. MIS-C involved the gastrointestinal, cardiovascular, hematologic, mucocutaneous, and respiratory systems requiring hospital admission, intensive care, mechanical ventilation, inotropic support, and extracorporeal membrane oxygenation (ECMO) [1, 2, 4]. The manifestation of the severe inflammatory condition resembled Kawasaki disease and hence called Kawasaki-like disease [5]. The etiology and pathogenesis remain unclear and are still being studied [1, 2, 5]. Owing to the phenotypic similarity of MIS-C with Kawasaki disease, similar treatment strategies to Kawasaki disease with IVIG alone or in combination with glucocorticoids have been used currently [1, 2, 6]. However, consensus/guideline to treat MIS-C is still unavailable.

We conducted our study to compare immunomodulation therapy with IVIG alone or IVIG with glucocorticoids and determine the clinical outcome in terms of treatment failure, need of adjunctive therapy, risk of left ventricular (LV) dysfunction, and need for inotropic treatment. In this systematic review and meta-analysis, we have reviewed the literature consolidating the best available evidence on the use and efficacy of IVIG alone or IVIG with glucocorticoids and report the superior treatment to put forward stronger evidence.

## 2. Methods

### 2.1. Information Source and Search Strategy

This study was done in adherence with the Preferred Reporting Items for Systematic Reviews and Meta-Analyses (PRISMA) statement [7]. We performed a systematic search of different electronic databases (MEDLINE via PubMed, EMBASE, and Google Scholar) and other sources which included Cochrane library and Clinicaltrials.gov. Secondary search was performed using articles retrieved from the primary search. Our comprehensive search strategy used the following MeSH terms and keywords: “MIS-C,” “COVID-19,” “coronavirus disease,” “coronavirus disease-19,” “severe acute respiratory syndrome,” and “therapeutics.” Studies published within the time frame of January 1, 2020, to August 27, 2021, were included for analysis. Search strategy for MEDLINE can be accessed in the S1 supplementary file.

### 2.2. Eligibility Criteria

We developed inclusion and exclusion criteria for the studies as follows.

#### 2.2.1. Inclusion Criteria

  Children aged less than 21 years
  Diagnosis of MIS-C based on the Center of Disease Control and Prevention (CDC) and World Health Organization (WHO) guideline
  All prospective or cross-sectional studies published in the English language, comparing IVIG versus IVIG plus glucocorticoids in MIS-C associated with COVID-19
  Studies assessing outcomes of mortality benefit, treatment failure, duration of PICU stay, need of adjunctive therapy, composite cardiovascular dysfunction, risk of left ventricular dysfunction, and need for inotropic treatment

#### 2.2.2. Exclusion Criteria

  Case reports, systematic review and meta-analysis, editorials, viewpoints, commentaries, missing/insufficient data, and irretrievable (articles in other languages and nonaccessible) studies were excluded

Based on the aforementioned criteria, title and abstract screening were done by two independent reviewers (Robin Rauniyar (RR1) and SG) using Covidence. The third reviewer (AM) reviewed all the studies of conflict. The full-text review of studies qualifying inclusion criteria after the title and abstract screening was done by another reviewer (Rohit Rauniyar (RR2) and SY). In case of conflict, another reviewer (GC) was sought to resolve the conflict.

### 2.3. Data Extraction

Two authors (RR1 and SK) independently extracted the data in MS Excel version 2016. An Excel sheet template was made, and data were extracted under the following headings: author name, study year, number of participants, and study design. Under each study, IVIG plus glucocorticoids and IVIG alone arms were made and outcomes of interest were extracted and recorded. The outcomes of interest that were extracted from the studies are treatment failure/persistence of fever, need for adjunctive therapy, risk of left ventricular dysfunction, and need for inotropes. Persistence of fever was taken as presence of fever at any point from day two or recrudescence after seven days of initial therapy. The need for adjunctive therapy was defined as the addition of a separate immunomodulator or an increment of 5 mg/kg or equivalent in the daily dose of prednisolone. LV dysfunction was defined as LVEF <55% on echocardiography. The need for inotropes was defined as an addition of an inotrope or escalation in the dose of previously prescribed inotrope. The extracted data were again reviewed by another reviewer (RR2).

### 2.4. Assessment of Methodological Quality

The quality of studies was assessed using the Newcastle–Ottawa scale (https://www.ohri.ca/programs/clinical_epidemiology/oxford.asp) for cohort studies by two reviewers (AM and SG). Three domains under the headings selection, comparability, and outcomes were used to give the scores. The mean score of greater than or equal to 5 was included in our study.

### 2.5. Data Analysis

Pooled proportions, odds ratio (OR), and 95% confidence intervals of outcomes of interest were generated from included studies. Forest plots of comparative ORs (IVIG plus glucocorticoids versus IVIG) were created using the STATA v16 software. Analyses were conducted using the random-effects model (DerSimonian-Laird analysis) if *I*^2^ > 50%, and the fixed-effects model was used if *I*^2^ < 50% [8]. Heterogeneity between trials was assessed using above models by *I*^2^ statistic [9]. Since the included study are few in number, the publication bias was not calculated for the outcomes and funnel plots was not prepared (Cochrane Handbook). The *p* value of <0.05 was considered statistically significant.

## 3. Results

### 3.1. Study Selection and Characteristics

**Figure 1:**
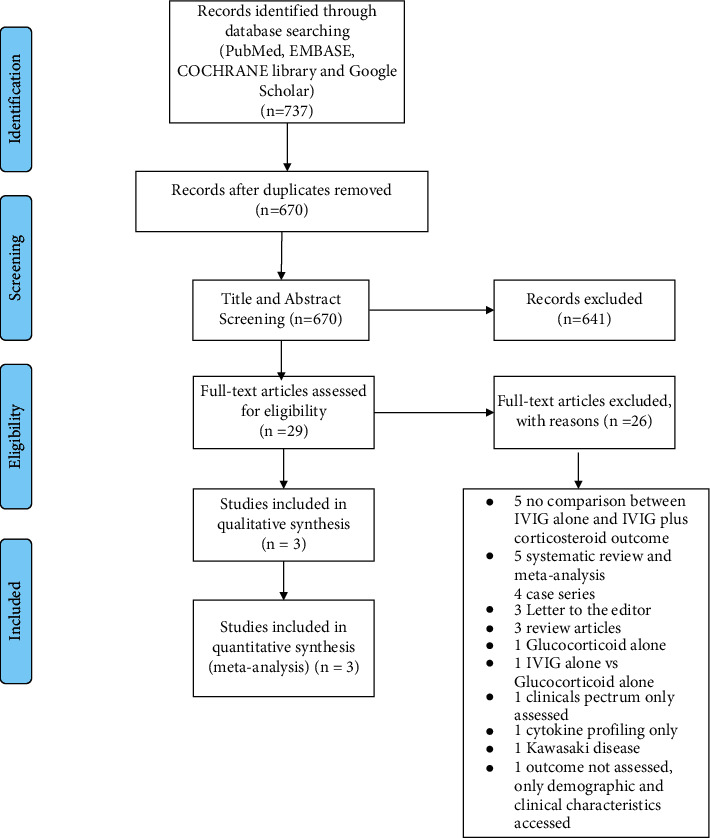
PRISMA flowchart showing selection of studies.

A total of 737 studies were identified through our primary database and secondary search. After the removal of 67 duplicates, 670 studies were selected for the title and abstract screening based on the inclusion criteria. 29 studies were eligible for full-text review and 3 studies were included in our final quantitative analysis [10–12]. The PRISMA flow diagram depicts the study retrieval process used (Figure 1).

The total number of patients in the included studies (*n* = 3) of our article was 756. The studies used propensity score matching or inverse weighted analysis for the reduction of bias. The total number of patients after propensity score matching in the IVIG plus glucocorticoid group was 343 and in the IVIG alone group was 413. All of the included studies were published in 2021 (Table 1).

### 3.2. Persistence of Fever/Treatment Failure

**Figure 2:**
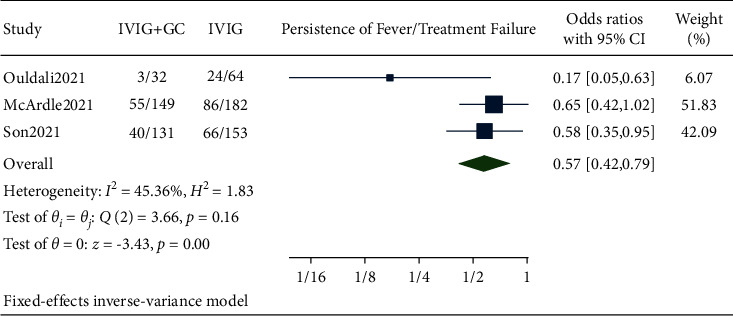
Forest plot showing the comparison of treatment failure/persistence of fever in IVIG plus glucocorticoids versus IVIG.

Pooled data from our studies showed persistence of fever or treatment failure in 98 out of 312 patients in the IVIG plus glucocorticoids group and 176 out of 399 patients in the IVIG group. The pooled results showed that the treatment failure was significantly lower in the IVIG plus glucocorticoids group compared to IVIG alone (OR 0.57, 95% CI (0.42, 0.79), *I*^2^ 45.36%, *p* < 0.05) (Figure 2).

### 3.3. Adjunctive Immunomodulatory Therapy

**Figure 3:**
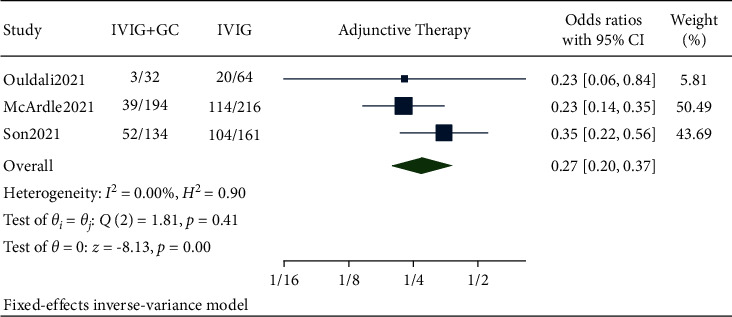
Forest plot showing the comparison of need of adjunctive therapy in IVIG plus glucocorticoids versus IVIG.

Pooled data from our studies showed the need for adjunctive immunomodulatory therapy in 94 out of 360 patients in the IVIG plus glucocorticoids group and 238 out of 441 patients in the IVIG group. The pooled results showed that the need for adjunctive immunomodulatory therapy was significantly lower in the IVIG plus glucocorticoids group compared to IVIG alone (OR 0.27, 95% CI (0.20, 0.37), *I*^2^ 0.0%, *p* < 0.05) (Figure 3).

### 3.4. Left Ventricular Dysfunction

**Figure 4:**
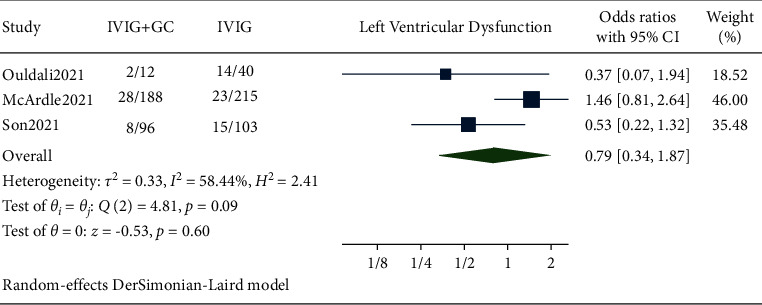
Forest plot showing the comparison of risk of left ventricular dysfunction in IVIG plus glucocorticoids versus IVIG.

Pooled data from our studies showed the occurrence of LV dysfunction in 38 out of 296 patients in the IVIG plus glucocorticoids group and 52 out of 358 patients in the IVIG group. The pooled results showed no significant reduction in the occurrence of left ventricular dysfunction in the IVIG plus glucocorticoids group compared to IVIG alone with *p* value >0.05 (OR 0.79, 95% CI (0.34, 1.87), *I*^2^ 58.44%) (Figure 4).

### 3.5. Need for Inotropes

**Figure 5:**
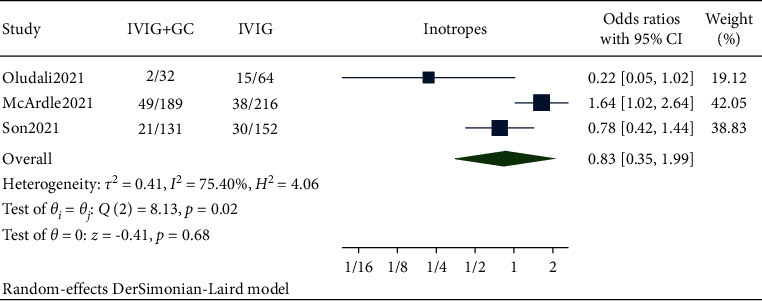
Forest plot showing the comparison of need of inotropic support in IVIG plus glucocorticoids versus IVIG.

Pooled data from our studies showed the need for inotropic treatment in 72 out of 352 patients in the IVIG plus glucocorticoids group and 83 out of 432 patients in the IVIG group. The pooled results showed that there is no significant reduction in the need for inotropes in the IVIG plus glucocorticoids group compared to IVIG alone with *p* > 0.05 (OR 0.83, 95% CI (0.35, 1.99), *I*^2^ 75.40%) (Figure 5).

### 3.6. Quality Assessment

The Newcastle–Ottawa scale done for cohort studies showed scores ranging from 5 to 6. All the studies greater than or equal to five were included in the analysis (Table 2).

## 4. Discussion

MIS-C is a hyperinflammatory condition seen in children whose spectrum of manifestation ranges from mild to severe form. It is characterized by fever, abdominal symptoms, cardiovascular symptoms, conjunctivitis, and rash. Typically seen after three to four weeks of COVID-19 infection, the disease can progress rapidly into severe shock and cardiorespiratory failure and further to death [13]. The management depends upon the severity of illness and clinical findings. The mainstay of treatment is the administration of IVIG; however, patients with mild symptoms may only need close monitoring. Other forms of immunomodulatory therapies such as glucocorticoids and anakinra are added based on the initial response and clinical manifestation. Concomitant administration of IVIG and glucocorticoid can be given in moderate to severe disease [14]. But studies comparing the efficacy of IVIG and IVIG with glucocorticoid are limited and are solely based on observational cohort studies.

Our meta-analysis conducted based on the existing data did support the use of combination therapy rather than IVIG alone for reducing treatment failure rate/persistence of fever. We found out that odds of having a treatment failure rate was lower in the combination group than in IVIG alone, which was found to be statistically significant. All the individual cohort studies included in the analysis also reported less likelihood of persistent fevers in combination therapy, though this was significant statistically only in the French study [10]. Although studies comparing the persistence of fever in both groups have not been done for MIS-C, treatment suggestions can be taken from such studies in Kawasaki disease as both of these entities show considerable phenotypic overlap. Combination therapy (IVIG plus glucocorticoids) also significantly reduced the duration of fever in Kawasaki disease [15].

Immunomodulatory therapies such as anakinra, infliximab, and tocilizumab are adjunctive or alternative treatment modalities in patients who show a stunted response to IVIG and steroid [16, 17]. All individual studies showed the reduced need for adjunctive immunomodulatory therapy [10, 11]. Similar result was obtained from the pooled analysis where combination therapy decreased the need for adjunctive immunomodulatory therapy with a significant *p* value without heterogeneity. A recent article about the single-center experience of cardiac outcomes in MIS-C suggested that the use of IVIG and steroid combination was associated with reduction in time to recovery of left ventricular ejection fraction [6]. Similar to this article, our analysis also found out that the occurrence of left ventricular dysfunction was lower in the combination therapy group. But this association was nonsignificant. A random effect model was used considering the high heterogeneity. Two included studies [10, 11] showed that the patients who received combination therapy were less likely to have left ventricular dysfunction one to two days after initial treatment. A systematic review reported that inotropes were given to 55.3% of the patients, similarly a study by Belhadjer et al. [18] also showed that the majority of MIS-C patients (80%) were under inotropic medications. The rationale for use of inotropes in MIS-C is its role in hemodynamic support. Two out of three included studies [10, 11] in our analysis showed the patients receiving combination therapy were less likely to need inotropic support than in IVIG alone. The pooled analysis showed a statistically nonsignificant reduction in the need for inotropes while using combination therapy rather than IVIG alone as a primary treatment.

Through our review and meta-analysis, we tried to quantitatively synthesize the available propensity score-matched data from observational cohort studies regarding appropriate management of MIS-C associated with COVID-19. This study provides the best available evidence regarding the efficacy of IVIG plus glucocorticoids when compared to IVIG alone in MIS-C studying various outcomes. Our study supports the use of combination therapy of IVIG plus glucocorticoids as it significantly lowered the risk of treatment failure or persistence of fever and the need for adjunctive immunomodulatory therapy in such patients. Our analysis also showed the lower risk of left ventricular dysfunction and decreased need for inotropic support with the use of combination therapy that was not statistically significant. Hence, the initial combination therapy with IVIG plus glucocorticoids seems to be the appropriate choice for the treatment of MIS-C compared to IVIG alone, although there is a dire need for randomized controlled trials that directly compare the two groups with appropriate outcomes.

## 5. Strength and Limitation

The major strength of our study is that this is the only meta-analysis to combine and quantify the evidence from the cohort studies comparing similar efficacy outcomes. The major limitation of our analysis is that the included studies are observational studies, and no randomized controlled trials, which would have yielded better results. Another major limitation is the difference in selection criteria of patients among different studies (WHO criteria versus CDC criteria for MIS-C). Other limitations include the availability of limited studies and the use of propensity score matching and inverse probability weighting among different studies for reduction of bias. Similarly, in a few numbers of studies, confounding factors such as age and gender affect the final combined output making the result unreliable with bias.

## Figures and Tables

**Table 1:** Baseline characteristics of the included studies.

Study	Year	No. of patients	Intervention	No. of patients	Age	Male sex (%)	Outcomes
Ouldali et. al.	2021	98	IVIG + GC	32	9.1 (4.7–13.1)	53	Treatment failure, adjunctive therapy, inotropic support, LV dysfunction, and duration of PICU stay
			IVIG	64	8.7 (4.6–12)	48	
McArdle et. al.	2021	454	IVIG + GC	208	8.8 (4.6–12)	61	Ventilated or death, improvement, treatment escalation, fever, death, any deterioration, LV dysfunction, and aneurysm
			IVIG	246	7.0 (3.7–11)	64	
Son et. al.	2021	206	IVIG + GC	103	8.8 (3.6–12)	56	Cardiovascular dysfunction, LV dysfunction, inotrope use, adjunctive therapy, and persistence of fever
			IVIG	103	7.6 (5.4–12.6)	57	

**Table 2:** Quality assessment of included cohort studies.

Study name	Selection	Comparability	Outcome	Total score	Included/excluded
Ouldali et. al.	3	2	1	6	Included
McArdle et. al.	2	2	1	5	Included
Son et. al.	2	2	1	5	Included

Mean scores greater than or equal to 5 are included in the analysis.

## Data Availability

The data used to support the findings of this study are available from the corresponding author upon request.

## Supplementary Materials

Supplementary Materials S1 supplementary file: search strategy.
