# Supplementary material for: IVIG plus Glucocorticoids versus IVIG Alone in Multisystem Inflammatory Syndrome in Children (MIS-C) Associated with COVID-19: A Systematic Review and Meta-Analysis
Source: Can J Infect Dis Med Microbiol. 2022 Mar 29;2022:9458653. doi: 10.1155/2022/9458653 (PMC8965733; doi:10.1155/2022/9458653)
Supplement: Supplementary Materials — S1 supplementary file: search strategy. [file 9458653.f1.docx]

**Search strategy for PubMed**

(((multisystem[All Fields] AND inflammatory[All Fields] AND ("syndrome"[MeSH Terms] OR "syndrome"[All Fields])) AND ("child"[MeSH Terms] OR "child"[All Fields] OR "children"[All Fields])) AND ("covid-19"[MeSH Terms] OR "covid-19"[All Fields] OR "covid19"[All Fields])) AND ("therapy"[Subheading] OR "therapy"[All Fields] OR "treatment"[All Fields] OR "therapeutics"[MeSH Terms] OR "therapeutics"[All Fields])

Total studies: 540

**Search strategy for Embase**

Embase

Session Results

.......................................................

No. Query Results Results Date

#15. #1 AND #11 AND #12 AND [humans]/lim AND 177

[27-08-2020]/sd NOT [28-08-2021]/sd AND

[2021-2022]/py

#14. #1 AND #11 AND #12 AND [humans]/lim 491

#13. #1 AND #11 AND #12 492

#12. #2 OR #5 9,279,043

#11. #6 AND #10 995

#10. #7 AND #9 54,925

#9. #3 OR #4 OR #8 320,677

#8. 'systemic inflammatory response syndrome'/exp 318,644

#7. 'child'/exp 3,142,043

#6. 'pediatric multisystem inflammatory syndrome'/exp 1,342

#5. 'therapy' 9,277,814

#4. 'mis c' 1,164

#3. misc 3,094

#2. 'treatment'/exp 5,836

#1. 'coronavirus disease 2019'/exp 190,970

**.......................................................**

Total studies:177

**Search strategy for Google Scholar**

**(**IVIG) and (Glucocorticoid) AND (MISC) AND (covid19)

Total studies: 20

**Search Strategy for Cochrane**

(IVIG and glucocorticoid):ti,ab,kw AND (glucocorticoid):ti,ab,kw AND (COVID19):ti,ab,kw AND (multisystem inflammatory syndrome):ti,ab,kw

Total studies=0

**Reasons for exclusion**
